# Characterization of a novel cysteine protease in *Trichinella spiralis* and its role in larval intrusion, development and fecundity

**DOI:** 10.1186/s13567-021-00983-1

**Published:** 2021-08-26

**Authors:** Yuan Yuan Hu, Ru Zhang, Shu Wei Yan, Wen Wen Yue, Jia Hang Zhang, Ruo Dan Liu, Shao Rong Long, Jing Cui, Zhong Quan Wang

**Affiliations:** Department of Parasitology, Medical College, Zhengzhou University, Zhengzhou, 450052 China

**Keywords:** *Trichinella spiralis*, cysteine protease, intrusion, fecundity, dsRNA

## Abstract

The aim of this study was to investigate the biological properties of a novel gut-specific cysteine protease in *Trichinella spiralis* (TsGSCP) and its role in larval intrusion, development and fecundity. TsGSCP has a functional C1 peptidase domain; C1 peptidase belongs to cathepsin B family. The TsGSCP gene cloned and expressed in *Escherichia coli* BL21 showed intensive immunogenicity. qPCR and Western blotting revealed that TsGSCP mRNA and protein were expressed at various *T. spiralis* stages, but their expression levels in intestinal infectious larvae (IIL) were clearly higher than those in muscle larvae (ML), adult worms (AWs) and new-born larvae (NBL). Indirect immunofluorescence (IIF) analysis showed that TsGSCP was primarily located at the outer cuticle and the intrauterine embryos of this parasite. rTsGSCP showed the ability to specifically bind with IECs, and the binding site is within the IEC cytoplasm. rTsGSCP accelerated larval intrusion into host intestinal epithelial cells (IECs), whereas anti-rTsGSCP antibodies suppressed larval intrusion; the acceleration and suppression was induced by rTsGSCP and anti-rTsGSCP antibodies, respectively, in a dose-dependent manner. When ML were transfected with TsGSCP-specific dsRNA, TsGSCP expression and enzymatic activity were reduced by 46.82 and 37.39%, respectively, and the capacity of the larvae to intrude into IECs was also obviously impeded. Intestinal AW burden and adult female length and fecundity were significantly decreased in the group of mice infected with dsRNA-transfected ML compared to the control dsRNA and PBS groups. The results showed that TsGSCP plays a principal role in gut intrusion, worm development and fecundity in the *T. spiralis* lifecycle and might be a candidate target for vaccine development against *Trichinella* intrusion and infection.

## Introduction

Trichinellosis is a worldwide zoonotic parasitic disease that is principally caused by the consumption of raw or undercooked meat containing *Trichinella* muscle larvae (ML) [1]. Mammals, rodents, amphibians, reptiles and birds are hosts of *Trichinella* [2]. Human *Trichinella* infection mainly results from eating infected pork in developing countries [3–5]. Twelve outbreaks of human trichinellosis due to infected pork or pork products were documented in China in 2004–2009 [6]. Because pork from domestic pigs is consumed in high quantities around the world, porcine *Trichinella* infection is a major risk to public health and meat safety [7]. Hence, it is necessary to develop preventive vaccines to interdict the transmission of swine *Trichinella* infection and eliminate *Trichinella* infectious larvae in animals consumed as food [8].

Following ingestion, *T. spiralis* ML encapsulated in host skeletal muscles are released from collagen capsules in the stomach by the action of digestive enzymes and activated into intestinal infective L1 larvae (IIL1) after exposure to intestinal contents or bile 0.9 h post-infection (hpi) [9, 10]. IIL1 recognize, invade and migrate within intestinal epithelium cells (IECs) and establish an intramulticellular niche in the intestinal columnar epithelium, where at 10 hpi IIL1 undergo their first moulting and develop into IIL2 and then moult three times before becoming adult worms (AWs) 10–31 hpi. After female and male AWs mate, pregnant adult females generate the next generation of new-born larvae (NBL), and these NBL pass into the blood circulation, invade skeletal muscles and are encapsulated to complete the lifecycle [11]. Invasion of IECs by IIL1 is the most important step in *T. spiralis* infection and parasitism within the host intestine. The intestinal epithelium is the first natural barrier against *T. spiralis* invasion and the main interaction site of the nematode and host [12, 13], but the mechanism of IIL1 penetration into IECs has not been fully elucidated [14, 15]. Identification and characterization of IIL1 invasion-related protein molecules in *T. spiralis* will contribute to an understanding of the IEC invasion mechanism and will be valuable to exploit preventive vaccines that block *Trichinella* invasion [16, 17].

Previous studies revealed that when they were cocultivated with an IEC monolayer, IIL invaded the monolayer and produced additional cysteine proteases that entered the IECs [18, 19]. Cysteine proteases have been identified in somatic soluble and excretory/secretory (ES) proteins of *T. spiralis* [20]. These cysteine proteases might promote IIL1 penetration of IECs in the process of *Trichinella* infection [21–23]. Our previous studies showed that several *T. spiralis* cysteine proteases participate in IIL1 invasion, and immune serum against recombinant cysteine proteases or RNAi partially suppressed IIL1 intrusion [24, 25]. Recombinant protein vaccination of mice led to only partial immune protection against *T. spiralis* challenge [26, 27]. These results suggested that other cysteine proteases participate in larval invasion of IECs. Therefore, it is necessary to characterize other cysteine proteases and evaluate their roles in larval intrusion, development and fecundity in the lifecycle of *T. spiralis*.

Cysteine proteases constitute a superfamily of widespread proteolytic enzymes in parasitic organisms and play a primary role in the lifecycle of parasites, including worm intrusion, migration and development, exsheathing, digestion and degradation of host proteins, and immune evasion [28–30]. The cysteine protease contains cathepsin B, C, F, H, K, L, O, S, V, W and X [31]. Cathepsin B is a member of the papain-like cysteine protease family from clan CA, exhibits conspicuous hydrolytic activity and might be secreted into the intercellular matrix and bound to the cellular membrane.

In this study, a novel gut-specific cysteine protease of *T. spiralis* (TsGSCP, GenBank: XP_003377240.1) was obtained from the *T. spiralis* draft genome [32]. TsGSCP has a functional C1 peptidase (cathepsin B family) domain. The aim of this study was to assess the biological properties of TsGSCP and its role in *T. spiralis* invasion of host intestinal mucosa.

## Materials and methods

### Experimental animals, *Trichinella* and cells

Four-week-old female KM mice were purchased from the Henan Experimental Animal Center. The *Trichinella spiralis* isolate (ISS534) used in this experiment was obtained from a domestic pig in Nanyang, Henan Province [33]. IECs were isolated from the small intestine of normal BALB/c mice [9]. The negative control cells were C2C12 myoblasts obtained from mouse muscles [34]. The bacterial strain used in the experiment was *Escherichia coli* BL21.

### Collection of worms at various stages and antigen preparation

The carcasses of mice infected with *T. spiralis* were artificially digested 40 days post-infection (dpi) to obtain ML [35]. The IIL and AWs were collected from the small intestine at 6 hpi, 3 dpi and 6 dpi [36, 37]. NBL were acquired from 6-day-old females after culturing for 24 h [38]. The soluble somatic worm antigens in various *T. spiralis* stages (ML, IIL, AW and NBL) and IIL ES antigens were prepared as previously described [39, 40].

### Bioinformatics analysis and evolutionary tree construction

The full-length cDNA sequence of the TsGSCP gene was obtained from NCBI. Bioinformatics analysis software (TargetP, TMHMM, SMART, ProtParam, Swiss-Model, ProtScale, PSIPred and SignalP) was used to analyse and predict its physicochemical properties, such as signal peptide, subcellular localization, tertiary structure of TsGSCP and functional sites [17]. The amino acid sequences of TsGSCP were compared with the cysteine protease from other organisms with BioEdit software [30]. The GenBank accession numbers of the cysteine proteases from other organism are as follows: *T. nelsoni* (KRΧ27624.1), *Trichinella* sp. T8 (KRZ92878.1), *T*. *nativa* (OUC48635.1), *T*. *patagoniensis* (KRY19675.1), *Trichinella* sp*.* T6 (KRX85497.1), *T. papuae* (KRZ80814.1), *T*. *zimbabwensis* (KRZ19202.1), *T*. *pseudospiralis* (KRZ16984.1), *Trichuris suis* (KFD71793.1), *Brugia malayi* (XP_001898194.1), *Caenorhabditis elegans* (NP_506002.2), *Schistosoma mansoni* (XP_018651608.1), *Echinococcus granulosus* (XP_024356149.1), *Mus musculus* (NP_031824.1) and *Homo sapiens* (NP_001899.1). The phylogenetic tree analysis of TsGSCP was performed with MEGA 7.0 using the neighbour-joining (NJ) method [41].

### Cloning and expression of rTsGSCP

According to the coding sequence (CDS) of TsGSCP after the signal peptide sequence was removed, TsGSCP-specific primers containing the *BamHI* and *PstI* restriction sites (bold) were designed*.* The primer sequences were 5′-GC**GGATCC**GCCTACTACGAAGAGACATA-3′ and 5′-CG**CTGCAG**TTATATTCTTGCCATTCCAGCTA-3′. cDNA was extracted from the ML and used as a template to amplify the TsGSCP gene. The recombinant expression plasmid pMAL-c2x/TsGSCP was constructed and introduced into *Escherichia coli* BL21 (Novagen, USA) [41, 42]. rTsGSCP was induced with 1 mM isopropyl β-d-1-thiogalactopyranoside (IPTG) at 25 °C for 6 h and purified by affinity chromatography using amylose resin (New England Bio-labs, USA).

### Preparation of immune serum and an anti‑rTsGSCP IgG titre assay

Ten mice were subcutaneously immunized using 20 μg of rTsGSCP emulsified with complete Freund’s adjuvant. Booster immunization was administered three times with 20 μg of rTsGSCP emulsified with incomplete Freund’s adjuvant with 2-week intervals [43, 44]. Tail blood from immunized mice was collected two weeks after the final immunization, and anti-rTsGSCP serum was isolated. Anti-rTsGSCP IgG titres were measured using rTsGSCP-ELISA as previously described [45]. In brief, the plate was coated with 1 μg/mL rTsGSCP at 4 °C and incubated overnight. After washing with PBST, the plate was blocked with 5% skim milk and incubated with serial dilutions of immune serum for 2 h at 37 °C, followed by incubation with HRP-conjugated anti-mouse IgG for 1 h at 37 °C. Colour was developed with OPD (Sigma-Aldrich, USA) plus H_2_O_2_, and the reaction was terminated with 2 M H_2_SO_4._ The absorbance at 492 nm was measured with a microplate reader (Tecan Schweiz AG, Switzerland) [46, 47].

### Western blot analysis

Soluble crude antigens of *T. spiralis* obtained at various stages, IIL ES antigens and purified rTsGSCP were separated by 10% SDS-PAGE. The proteins were transferred onto nitrocellulose (NC) membranes (Millipore, USA) in semidry transfer cells (Bio-Rad, USA) [37, 48]. The membrane was blocked with 5% skim milk in Tris-buffered saline containing 0.05% Tween (TBST) at 37 °C for 2 h and cut into strips. The strips were probed by various sera (1:200; anti-rTsGSCP serum, infection serum and preimmune serum) at 37 °C for 2 h. Following washes with TBST, the strips were incubated at 37 °C for 1 h with HRP-conjugated anti-mouse IgG (1:10 000; Southern Biotech, USA). After being rewashed, the strips were coloured using 3,3′-diaminobenzidine tetrahydrochloride (DAB; Sigma-Aldrich, USA) or reagent from an enhanced chemiluminescent kit (CWBIO, Beijing, China) [49].

### qPCR assay

Total RNA from diverse *T. spiralis* phases (ML, IIL, 3- and 6-day AW, and NBL) was isolated using TRIzol reagent (Invitrogen, USA). TsGSCP mRNA expression levels at diverse worm phases were assessed using qPCR as described previously [10]. The TsGSCP-specific primers used for qPCR were 5′-ATGCGGCTATGGATGTGACGG-3′ and 5′-GTGTGCACAACGGTGTTTCAGC-3′. The relative level of TsGSCP mRNA expression was normalized by subtracting the mRNA expression level of the *T. spiralis* internal control gene tubulin (GenBank: XM_003369432.1) [50] and then calculated according to the comparative Ct (^2−ΔΔCt^) method [20]. Each experiment was performed with three replicates.

### Indirect immunofluorescence (IIF) analysis

IIF analysis was performed with whole worms and their cross sections as reported previously [51, 52]. Whole intact worms in different *T. spiralis* phases (ML, 6 and 12 h IIL, 3- and 6-day AWs, and NBL) were fixed with 4% paraformaldehyde at room temperature for 30 min, washed three times with PBS, and then immobilized in cold acetone at −20 °C for 20 min. Moreover, the ML, IIL and AWs were embedded in paraffin, and 2-µm-thick worm cross-sections were sliced with a microtome. Worm cross-sections were first blocked with goat serum and subsequently with 0.3% H_2_O_2_ at room temperature for 20 min. The parasites and cross-sections were probed with diverse sera (1:10; anti-rTsGSCP serum, infection serum, preimmune serum and anti-MBP-tag serum) at 37 °C for 2 h. Following washes in PBS, the worms and cross-sections were incubated with FITC-conjugated anti-mouse IgG (1:100; Abways, Shanghai, China). After a rewash, the whole worms and cross-sections were examined under fluorescence microscopy (Olympus, Japan) [53, 54].

### Far-Western blotting showing rTsGSCP binding with IEC proteins

IECs from the mouse intestine were sensitive to *T. spiralis* penetration, whereas mouse C2C12 cells from a subpopulation of striated muscle myoblasts were insensitive to penetration and served as negative control cells [34, 55]. The binding of rTsGSCP and IECs was ascertained by far-Western blotting. Soluble IEC proteins were separated by SDS-PAGE, transferred onto NC membranes, cut into strips and blocked with 5% skim milk. Following washes with PBST, the strips were incubated with 20 μg/mL rTsGSCP at 37 °C for 2 h, subsequently probed with anti-rTsGSCP serum and incubated with HRP-conjugated anti-mouse IgG (1:10 000, Southern Biotech). Finally, colour was developed with DAB (Sigma-Aldrich).

### Binding of rTsGSCP with IECs proteins as determined by ELISA

Binding of rTsGSCP and IEC proteins was assessed by ELISA as reported previously [45]. Briefly, a plate was coated with diluted IEC proteins (0.01, 0.1, 0.2, 0.3, 0.4, 0.5 and 0.6 μg/mL) at 4 °C overnight. After being blocked with 5% skim milk and washed using PBST, the plates were incubated with diluted concentrations of rTsGSCP (0.01, 0.5, 1, 1.5, 2, 2.5 and 3 μg/mL) at 37 °C for 2 h. Following washing, the plates were probed with anti-rTsGSCP serum (1:100) and incubated with HRP-conjugated anti-mouse IgG (1:10 000, Southern Biotech). Colour was developed using *o*-phenylenediamine dihydrochloride (OPD; Sigma-Aldrich), and absorbance at 492 nm was measured [56].

### IIF analysis of rTsGSCP and IEC binding

The binding of rTsGSCP and IECs and its cellular localization were evaluated using IIF [56]. IECs were cultivated in a 6-well culture plate until confluent [57]. Then, the IECs were incubated with rTsGSCP (20 μg/mL) at 37 °C for 2 h. Following washes, the IECs were fixed in cold acetone for 10 min and subsequently blocked with 5% goat serum at 37 °C for 2 h. IECs were incubated with anti-rTsGSCP immune sera (1:10 dilution) as the primary antibody. FITC-conjugated anti-mouse IgG (1:100; Abways) was used as the secondary antibody. Cell nuclei were stained with 4′,6-diamidino-2-phenylindole (DAPI) and examined by fluorescence microscopy [22].

### Larval intrusion into IECs in vitro

To investigate whether TsGSCP acts on larval intrusion into the intestinal epithelium, an in vitro intrusion assay was performed as previously described [9]. In brief, ML were first activated and transformed into IIL upon exposure to 5% mouse bile for 2 h at 37 °C. One hundred IIL were added to an IEC monolayer cultured in DMEM semisolid medium (DMEM supplemented with l-glutamine, 15 mM HEPES, and 1.75% agarose). The medium was presupplemented with various concentrations of rTsGSCP (1.5, 3, 6, 9, 12 and 15 μg/mL) or serially diluted anti-rTsGSCP serum (1:100–1:1000). After cultivation at 37 °C and 5% CO_2_ for 2 h, larval intrusion into the monolayer was observed by microscopy [48]. The invaded larvae were mobile and migrated into the monolayer, leaving a clear migratory trace, whereas noninvading larvae were coiled on the monolayer surface.

### RNAi analysis

The TsGSCP-specific dsRNA target sequence of the complete TsGSCP cDNA sequence was used to design the primers (Table 1) [58]. To confirm TsGSCP-dsRNA specificity, a *T. spiralis* aspartyl aminopeptidase (TsAAP, GenBank: KRY29491.1) was selected as a specific control. Additionally, green fluorescent protein (GFP) dsRNA was also prepared as a control. dsRNA was transfected into *T. spiralis* larvae by electroporation and cultured in RPMI 1640 medium at 37 °C for 2–6 days. qPCR and Western blotting were performed to assess TsGSCP expression in the ML as reported previously [59, 60], and the expression level of the *T. spiralis* housekeeping gene tubulin was used as the internal control. The protein expression level was assessed based on densitometry, and the results showed the relative protein expression as assessed in three repeated experiments [61].

**Table 1:** **TsGSCP-specific primers flanked by T7 RNA polymerase promoter sequences**

Primers	Sequences
T7-TsGSCP-F	5′-GATCACTAATACGACTCACTATAGGGGCTAGAAAACGATGGCCACA-3′
T7-TsGSCP-R	5′-GATCACTAATACGACTCACTATAGGGGCCATTCCAGCTACAACATA-3′
T7-GFP-F	5′-GATCACTAATACGACTCACTATAGGGTCCTGGTCGAGCTGGACGG-3′
T7-GFP-R	5′-GATCACTAATACGACTCACTATAGGGCGCTTCTCGTTGGGGTCTTTG-3′

The underlined portion is the T7 promoter sequence

The enzymatic activity of natural TsGSCP in crude soluble proteins of dsRNA-TsGSCP-transfected larvae was analysed and compared with that of untransfected larvae as previously described [22]. Briefly, ML and IIL were transfected with 40 ng/μL dsRNA-TsGSCP and cultured for 2 days, and somatic soluble proteins of dsRNA-treated ML and IIL were prepared. The enzymatic activity of native TsGSCP in worm crude proteins was ascertained using the cysteine protease-specific substrate Z-Phe-Arg-7-amido-4-methylcoumarin hydrochloride (Z-FR-AMC; Sangon, China).

### Challenge by infection with dsRNA-treated larvae in mice

To evaluate larval infectivity, survival and fecundity in the host intestine following dsRNA interference, 30 mice were assigned to 3 groups (10 animals per group). Each mouse was inoculated orally with 500 ML treated with 40 ng/μL dsRNA-TsGSCP, dsRNA-GFP or PBS. The infected mice were euthanized 5 dpi, adult worms were removed from the intestine, the adult worm burden was assessed, and adult worm morphology and size were examined by microscopy. Thirty adult females from each group of infected mice were cultured, and female reproductive ability (fecundity) was ascertained on the basis of NBL production by each female within 72 h [62].

### Statistical analysis

All the data were analysis with SPSS 21.0 software. The results are shown as the means ± standard deviation (SD). TsGSCP expression level, worm burden and length, and NBL production among different groups were analysed with one-way ANOVA. Chi-square test was performed to analyse the larval invasion rate and TsGSCP expression after RNAi in the various groups. *P* < 0.05 is considered to be statistically significant.

## Results

### Bioinformatics analysis of TsGSCP

**Figure 1:**
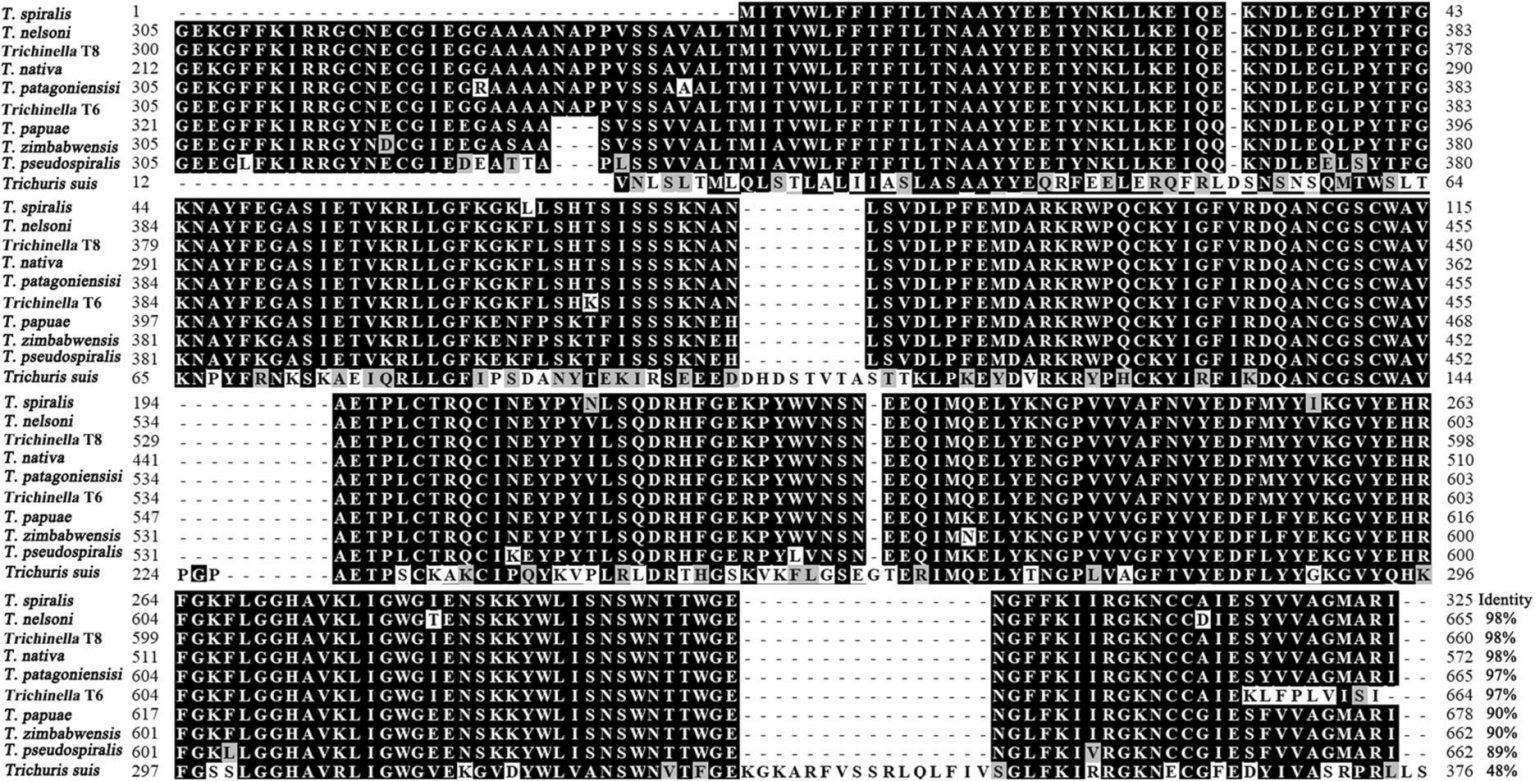
**Sequence alignment of TsGSCP.** Sequence alignment of the TsGSCP gene with genes in other nematodes. The black shading indicates residues that are the same in the TsGSCP gene, the grey shading indicates the residues that are conservatively substituted, and the end number of the sequence indicates the shared identity percentage of TsGSCP with cysteine protease of other nematodes.

**Figure 2:**
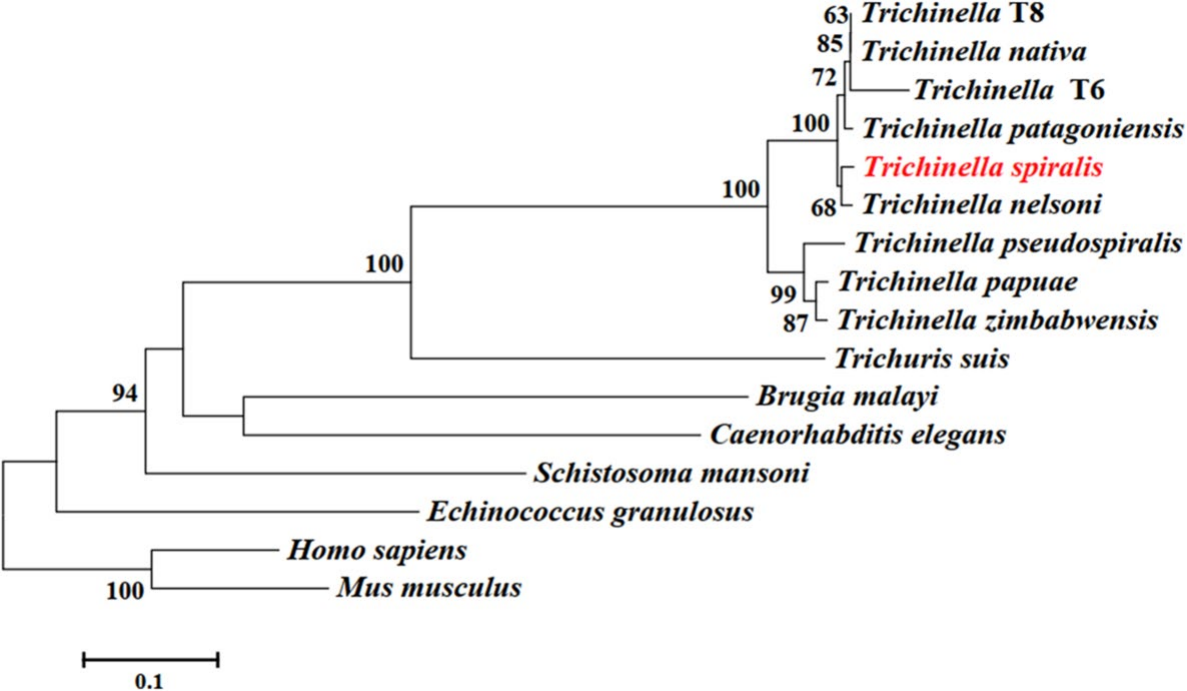
**Phylogenetic tree of cysteine proteases in 16 organisms as produced through the NJ method** (the red font represents TsGSCP).

The CDS of TsGSCP is 991 bp, encoding 325 amino acids with a relative molecular weight of 36.9 kDa and isoelectric point (pI) of 6.88. TsGSCP does not contain a transmembrane region, but it has a signal peptide. The subcellular location is a secretory pathway, indicating that the TsGSCP protein may be a secretory protein. The tertiary structure of TsGSCP has 4 active enzymatic sites (His, Gln, Cys and Asn). Using BioEdit software to compare the TsGSCP amino acid sequences in different *Trichinella* species, the following results were obtained: the amino acid sequence of TsGSCP shared 98% identity with cysteine protease of *T. nelsoni*, *Trichinella* T8 and *T. nativa*, 97% identity with those of *T. patagoniensis* and *Trichinella* T6, 90% identity with those of *T. papuae* and *T. zimbabwensis*, and 89% with *T. pseudospiralis* (Figure 1). The results indicated that TsGSCP is highly similar between encapsulated *Trichinella* species, suggesting that TsGSCP is not species-specific. A Phylogenetic tree analysis of TsGSCP and cysteine proteases in other organisms is shown in Figure 2. The phylogenetic tree showed that a monophyletic group of the genus *Trichinella* was well supported. Within the genus *Trichinella*, two clades were obvious: one was the encapsulated species clade (*T. spiralis*, *T. nelsoni*, *T. patagoniensis*, T6, *T. nativa*, and T8), and the other was the nonencapsulated species clade (*T. pseudospiralis*, *T. papuae* and *T. zimbabwensis*).

### Analyses of rTsGSCP expression and antigenicity

**Figure 3:**
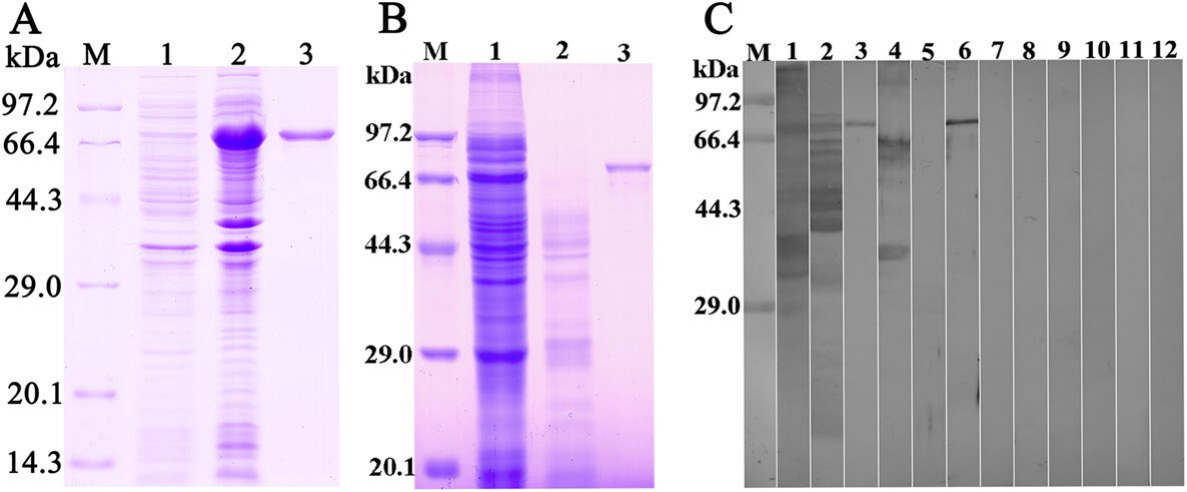
**Expression and antigenicity analyses of rTsGSCP. A** SDS-PAGE gel showing rTsGSCP. Lane M, protein marker; lane 1, lysate of bacteria carrying pMAL-c2x/TsGSCP prior to induction; lane 2, lysate of induced bacteria carrying pMAL-c2x/TsGSCP; lane 3, purified pMAL-c2x/TsGSCP. **B** SDS-PAGE gel showing of worm antigens. Lane M, protein marker; lane 1, IIL soluble crude proteins; lane 2, IIL ES proteins; lane 3, purified rTsGSCP. **C** Western blot analysis of rTsGSCP. IIL soluble protein (lane 1), IIL ES protein (lane 2) and rTsGSCP (lane 3) were recognized with infection serum. Natural TsGSCP in IIL crude proteins (lane 4) and rTsGSCP (lane 6) were identified with anti-rTsGSCP serum, but native TsGSCP was not found among IIL ES proteins (lane 5) when probed with anti-rTsGSCP serum. Crude IIL (lanes 7 and 10), ES proteins (lanes 8 and 11), and rTsGSCP (lanes 9 and 12) were not recognized by preimmune serum (lanes 7–9) or anti-MBP-tag serum (lanes 10–12).

In the SDS-PAGE analysis, purified rTsGSCP was identified as a clearly visible individual band, and its molecular weight (74.2 kDa) was consistent with its predicted size (rTsGSCP is 34.2 kDa, and the MBP-tag protein is 40 kDa) (Figure 3). The serum anti-rTsGSCP IgG titre was 1:10^5^ following the final immunization, demonstrating that rTsGSCP induced immunogenicity. The results of the Western blot analysis showed that rTsGSCP was recognized by anti-rTsGSCP serum and infection serum but not by anti-MBP-tag serum and preimmune serum. Furthermore, natural TsGSCP of 35.2, 49.6, 56.5, 61.6 kDa in IIL soluble proteins was identified by anti-rTsGSCP serum but was not found among IIL ES proteins, suggesting that TsGSCP is a somatic worm protein, not a secretory protein.

### TsGSCP mRNA and protein expression in different stages

**Figure 4:**
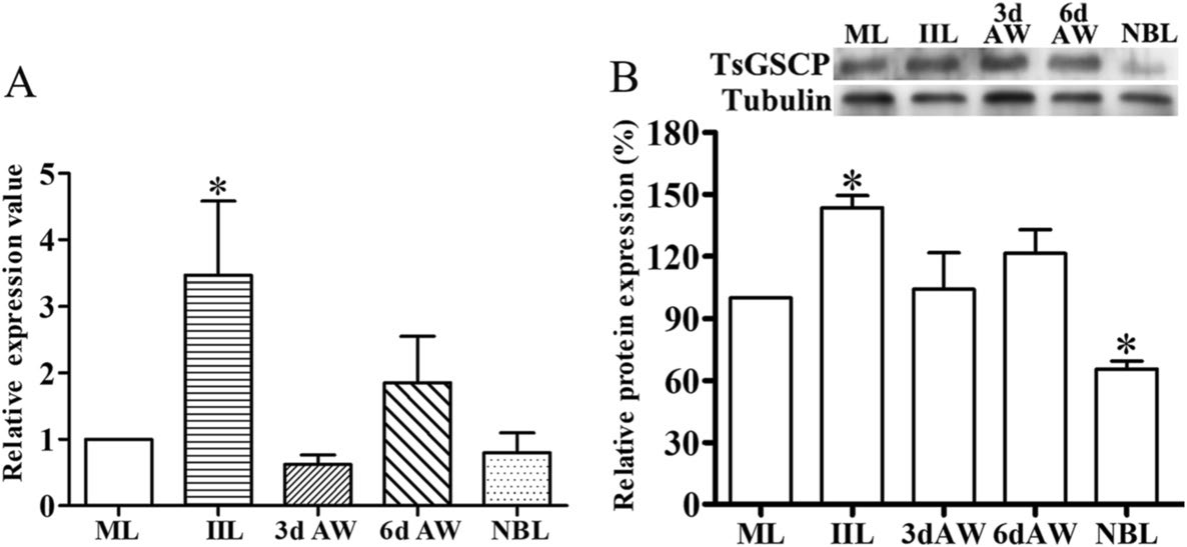
**TsGSCP transcription and expression in the lifecycle of *T. spiralis.* A** qPCR assay of TsGSCP transcription in different worm phases. **B** Western blot results showing TsGSCP expression in different stages based on three independent densitometry tests. The relative transcription and expression level of TsGSCP at the IIL stage was clearly higher than at other worm stages (*P < 0.05).

The qPCR results showed that TsGSCP transcription level in the IIL stage was significantly higher than that in the worms at other stages (*F* = 4.840, *P* < 0.05) (Figure 4A). Moreover, the TsGSCP expression level in the IIL stage was higher than that in the ML or AW stage (*F* = 7.778, *P* < 0.05) (Figure 4B). These results indicated that both TsGSCP mRNA and protein expression in the IIL stage was higher than that in the other developmental stages of the *T. spiralis* lifecycle, suggesting that TsGSCP might be a larva intrusion-related protein.

### Expression and tissue localization of natural TsGSCP in *T. spiralis*

**Figure 5:**
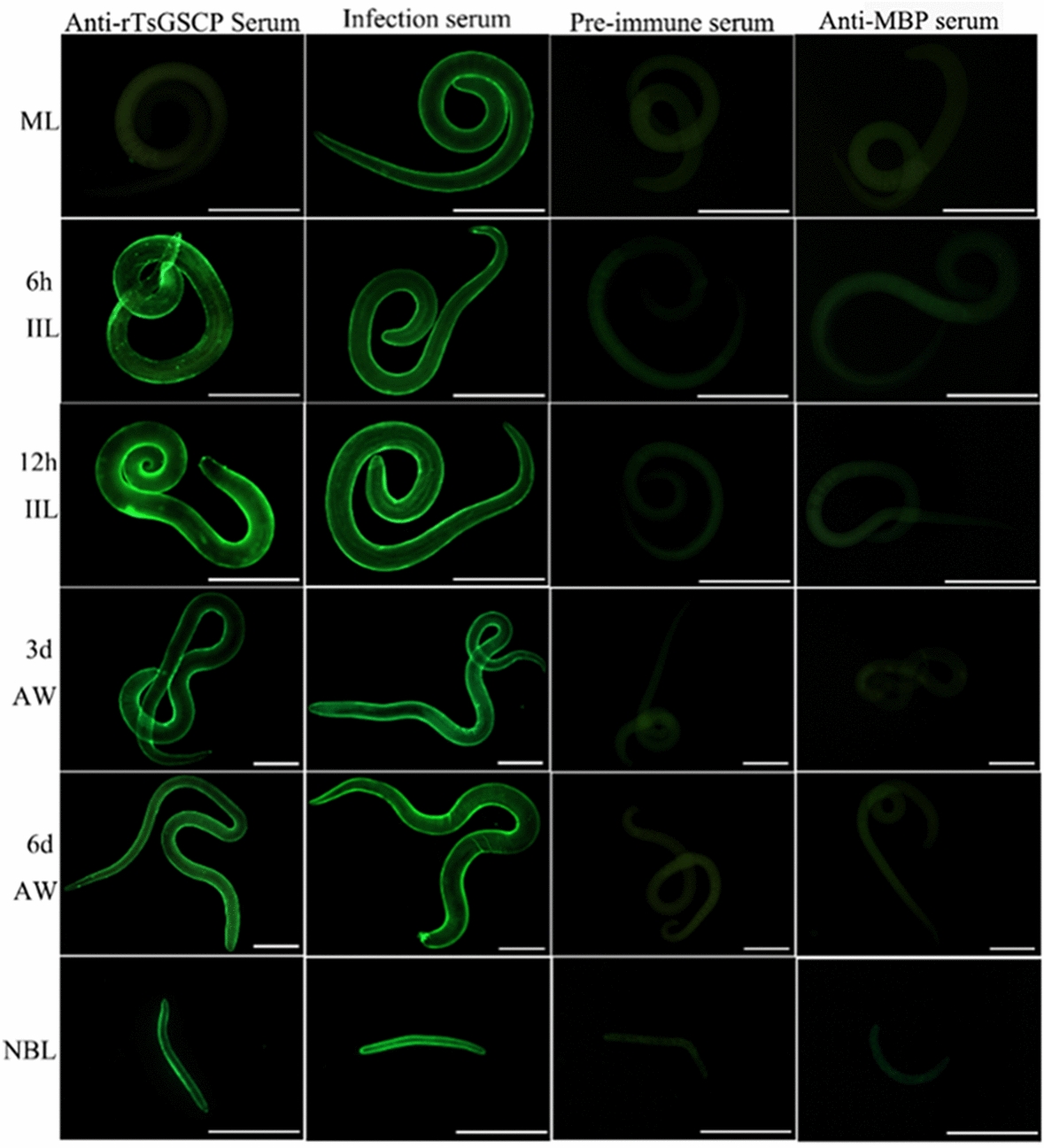
**Expression of TsGSCP on the cuticle of *T. spiralis* in different stages, as identified by IIF analysis with anti-rTsGSCP serum*.*** Bright fluorescence staining was observed on the outer surface of cuticles in worms at each stage except the muscle larva stage. The nematodes recognized with infection serum were used as positive controls; preimmune serum and anti-MBP-tag serum were used as the negative controls. Scale bar of ML and IIL = 50 μm, Scale bar of AWs = 100 μm, Scale bar of NBL = 25 μm.

**Figure 6:**
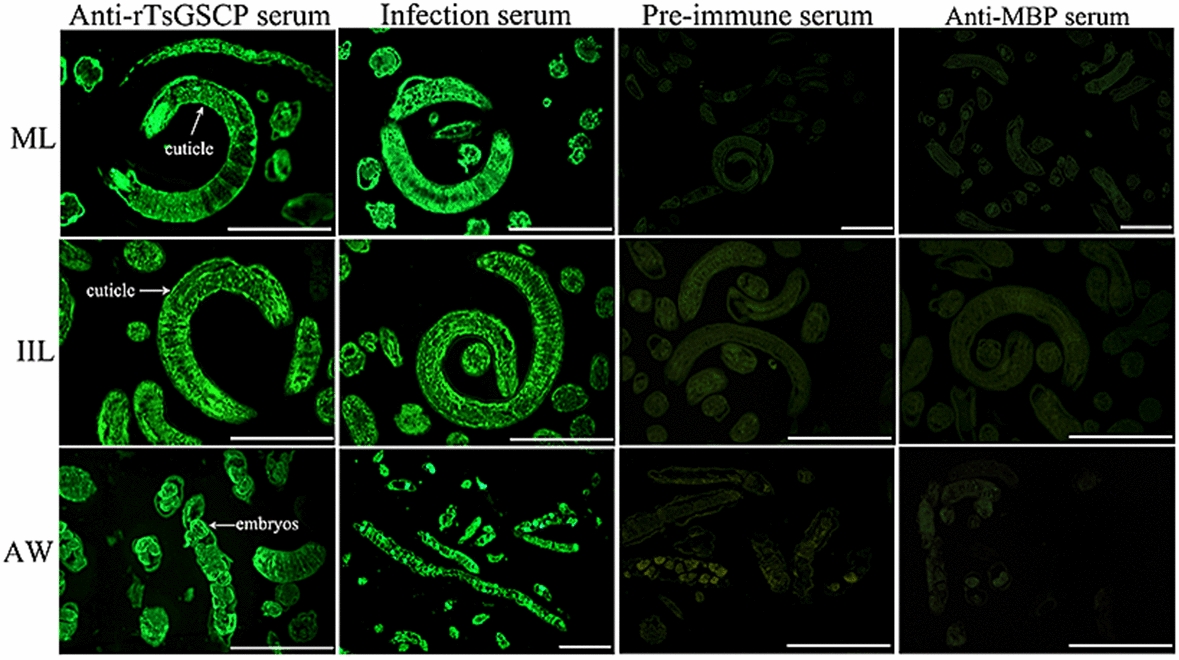
**Tissue localization of TsGSCP in *T. spiralis* in different stages.** IIF analysis with worm cross-sections was performed using anti-rTsGSCP serum. Immune staining was primarily localized in the cuticle of ML and IIL and female intrauterine embryos in the nematode. No immune staining in worm cross-sections was observed with preimmune serum or anti-MBP serum, which were the negative controls. Scale bars, 50 μm.

An IIF analysis was performed to assess the expression and localization of TsGSCP in *T. spiralis*. The results are shown in Figure 5. In anti-rTsGSCP serum, bright green fluorescence was observed on the cuticle surface of worms in each stage except those in the muscle larva stage. In the IIF analysis performed with worm cross-sections, the fluorescence staining was principally located in the cuticle of ML and IIL and female intrauterine embryos in the nematode (Figure 6).

### Binding of rTsGSCP and IEC proteins as assessed with far-Western blotting and ELISA

**Figure 7:**
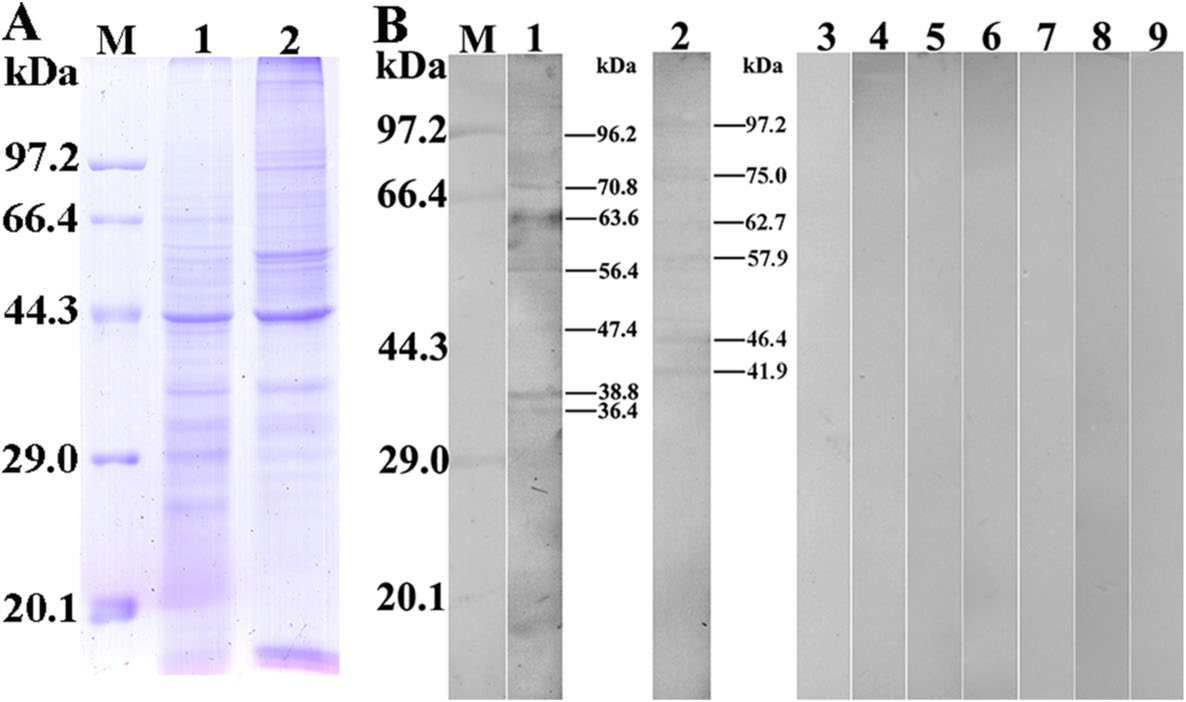
**Far-Western blot identification of binding between rTsGSCP and IEC proteins. A** SDS-PAGE analysis of soluble IEC proteins (lane 1) and C2C12 cell proteins (lane 2). Lane M, protein marker. **B** Far-Western blotting showing the binding of rTsGSCP with IEC proteins. Lane M, protein marker. The strips containing IEC protein (lanes 1–6) were incubated with rTsGSCP (lanes 1–3) or MBP (lanes 4–6). Bands of indicative of rTsGSCP binding with IECs were identified with anti-rTsGSCP serum (lane 1) and infection serum (lane 2) but not with preimmune serum (lane 3). No binding of MBP with IECs was detected with anti-MBP serum (lane 4), infection serum (lane 5) or preimmune serum (lane 6). There was no binding of rTsGSCP with C2C12 cells when anti-rTsGSCP serum (lane 7), infection serum (lane 8) or preimmune serum (lane 9) was used.

The SDS-PAGE results revealed that soluble IEC proteins separated into approximately 28 bands in a range between 20.1 and 97.2 kDa (Figure 7A). In far-Western blotting, after IEC proteins were incubated with rTsGSCP, 7 bands (96.2, 70.8, 63.6, 56.4, 47.4, 38.8 and 36.4 kDa) were identified with anti-rTsGSCP serum, and 6 bands (97.2, 75.0, 62.7, 57.9, 46.4 and 41.9 kDa) were detected with infection serum. No IEC proteins preincubated with rTsGSCP were identified with preimmune serum, and no C2C12 proteins preincubated with rTsGSCP were detected by anti-rTsGSCP serum or infection serum (Figure 7B). These results indicated that TsGSCP specifically binds to IEC proteins.

**Figure 8:**
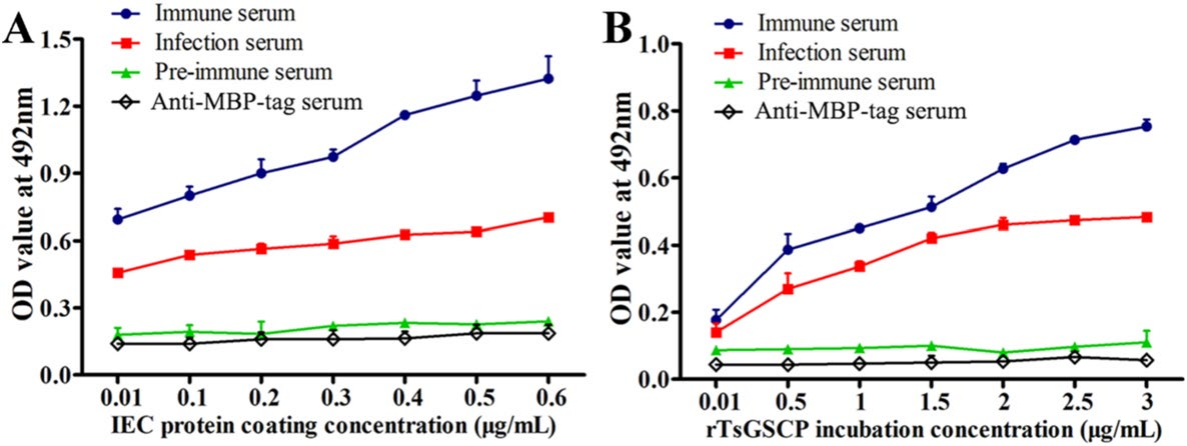
**Binding of rTsGSCP with IECs as detected by ELISA**. **A** Binding of IEC proteins with diluted coating and incubation with 1 μg/mL rTsGSCP. **B** Binding of 1 μg/mL IEC protein with coating and incubation with different doses of rTsGSCP. The binding between rTsGSCP and IEC proteins is dose-dependent.

The ELISA results showed an evident interaction of rTsGSCP with IEC proteins. The absorbance measure of the IEC proteins was dose-dependent (*r* = 0.994, *P* < 0.0001) and showed an increasing trend with increasing IEC protein coating concentration (*F* = 397.007, *P* < 0.0001) (Figure 8A). Moreover, the absorbance was rTsGSCP dose-dependent (*r* = 0.976, *P* < 0.0001) and showed a positive trend with increasing rTsGSCP dose (*F* = 99.967, *P* < 0.0001) (Figure 8B). The results demonstrated that rTsGSCP can bind to IEC proteins, which might play a major role in larval intrusion into IECs.

### Binding of rTsGSCP with IECs and its cellular localization

**Figure 9:**
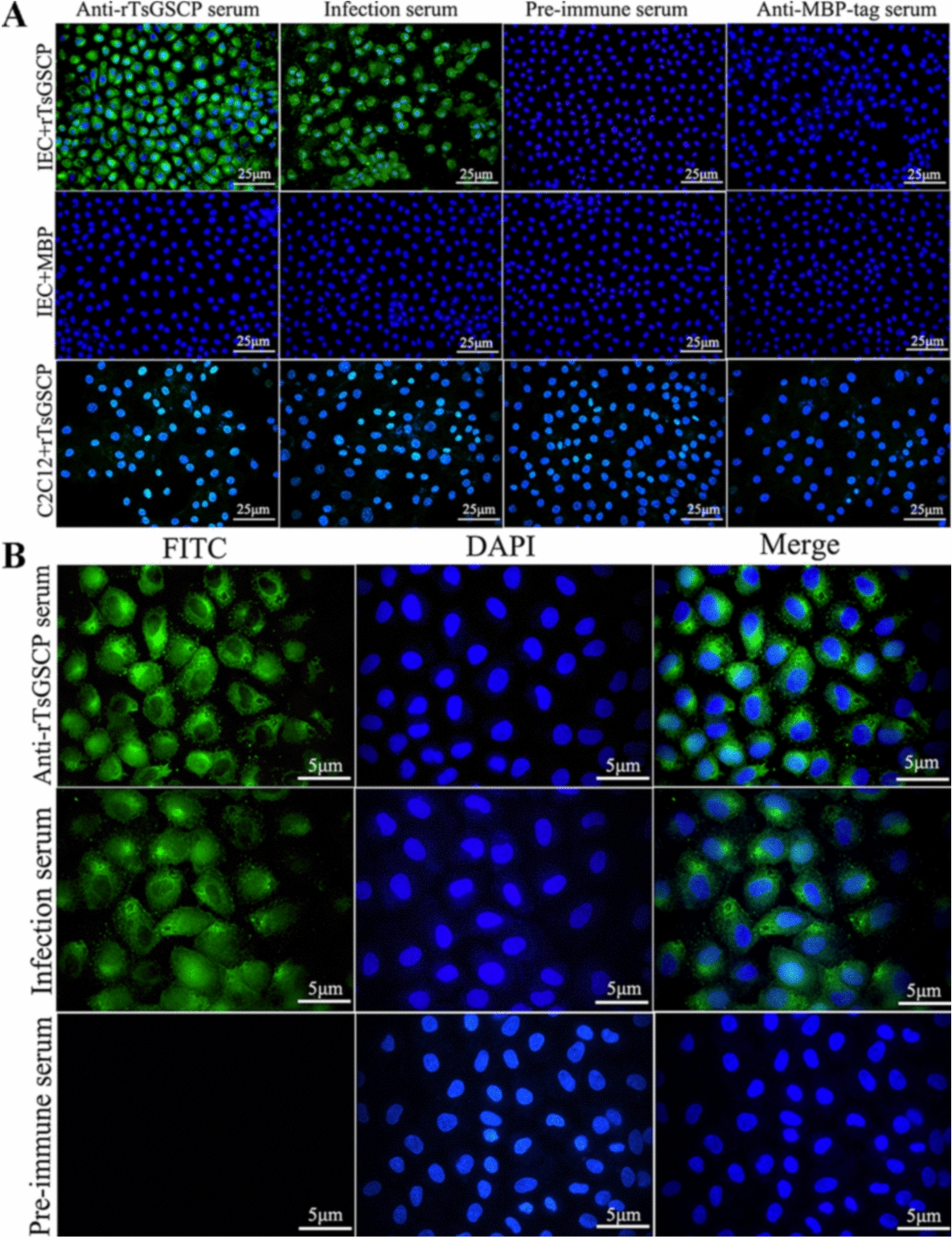
**Specific binding of rTsGSCP with IECs and cellular localization. A** IIF analysis showing the specific binding of rTsGSCP with IECs. IECs or C2C12 cells were preincubated with rTsGSCP or MBP. After blocking and washing, the cells were probed with anti-rTsGSCP serum, infection serum, anti-MBP-tag serum or preimmune serum, followed by incubation with FITC-conjugated anti-mouse IgG. Cell nuclei were stained blue with DAPI and examined by fluorescence microscopy (400 ×). Scale bars, 25 μm. **B** Cellular localization of rTsGSCP within IECs as shown at high magnification (1000 ×). The IECs were preincubated with rTsGSCP, subsequently incubated with anti-rTsGSCP serum, infection serum or preimmune serum, and finally stained with FITC-conjugated anti-mouse IgG. DAPI was applied to stain cell nuclei blue. Scale bars, 5 μm.

The IIF analysis results showed that after IECs were preincubated with rTsGSCP, green immunofluorescence staining was observed on the surface of the IECs probed with anti-rTsGSCP serum and infection serum but not with preimmune serum. The IECs preincubated with MBP alone did not show any immune staining upon treatment with anti-rTsGSCP serum or infection serum. No immune staining on the C2C12 surface was detected when C2C12 cells preincubated with rTsGSCP were probed with anti-rTsGSCP serum or infection serum (Figure 9A). At high magnification, immune staining was observed to be distributed in the cytoplasm of IECs (Figure 9B), suggesting that rTsGSCP has specific binding ability with IECs and enters the cytoplasm.

### rTsGSCP promotion and anti-rTsGSCP serum suppression of larval intrusion

**Figure 10:**
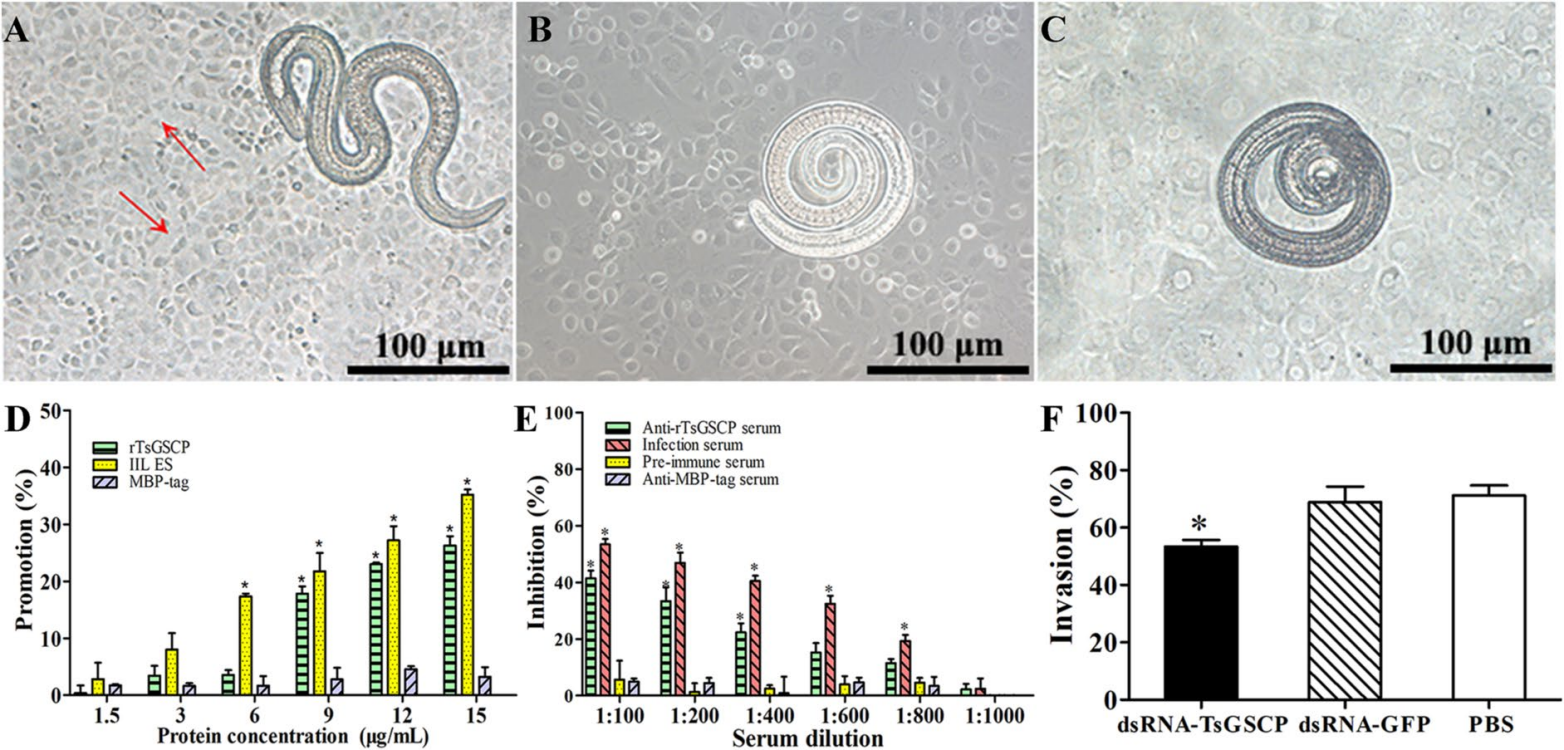
**Facilitation or inhibition of larval intrusion by rTsGSCP or anti-rTsGSCP serum.** IILl were added to an IEC monolayer, and invaded larvae were examined under a microscope 2 h after cultivation. **A** The invading larvae were mobile and migratory in the monolayer (the red arrow shows migratory trace); noninvading worms were coiled on the surface of the IECs (**B**) and C2C12 cells (**C**). **D** rTsGSCP accelerated worm intrusion. **E** Anti-rTsGSCP serum suppressed larval intrusion. **F**: dsRNA-TsGSCP inhibited larva invasion into IECs. *P < 0.0001 compared to the PBS control groups. Scale bars, 100 μm.

As shown in Figure 10A, invaded larvae left a clear migratory trace (red arrow), and uninvaded larvae were coiled on the monolayer surface (Figures 10B and C). After the medium was supplemented with rTsGSCP and IIL were cultured in this medium for 2 h, rTsGSCP significantly accelerated larval intrusion. This acceleration was rTsGSCP dose-dependent (*r* = 0.950, *P* < 0.0001) and exhibited an increasing trend with increasing rTsGSCP dose (*F* = 228.325, *P* < 0.0001), but MBP-tag did not facilitate intrusion (Figure 10D). When the medium was supplemented with different dilutions of anti-rTsGSCP serum, anti-rTsGSCP serum (1:100–1:400) significantly impeded larval intrusion relative to PBS (χ^2^ = 16.743, *P* < 0.0001). The suppression of IEC intrusion was dose-dependent for anti-rTsGSCP antibodies (*r* = 0.969, *P* < 0.0001), and the suppressive effect was reduced with increasing serum dilution (*F* = 136.542, *P* < 0.0001) (Figure 10E). Furthermore, the invasion rate of IECs with IIL treated with dsRNA-TsGSCP, dsRNA-GFP and PBS was 53.39, 68.9 and 71.2%, respectively (*P* < 0.05), and the invasive ability of the larvae treated with dsRNA-TsGSCP was inhibited by 25.02% compared to that of the dsRNA-GFP group (χ^2^ = 5.578, *P* < 0.05) (Figure 10F). These results suggested that TsGSCP promoted larval intrusion, whereas anti-rTsGSCP antibodies and silencing of the TsGSCP gene inhibited larval intrusion, indicating that TsGSCP plays an important role in larval intrusion into host gut epithelium during *Trichinella spiralis* infection.

### Reduction in TsGSCP expression and activity after TsGSCP gene silencing

**Figure 11:**
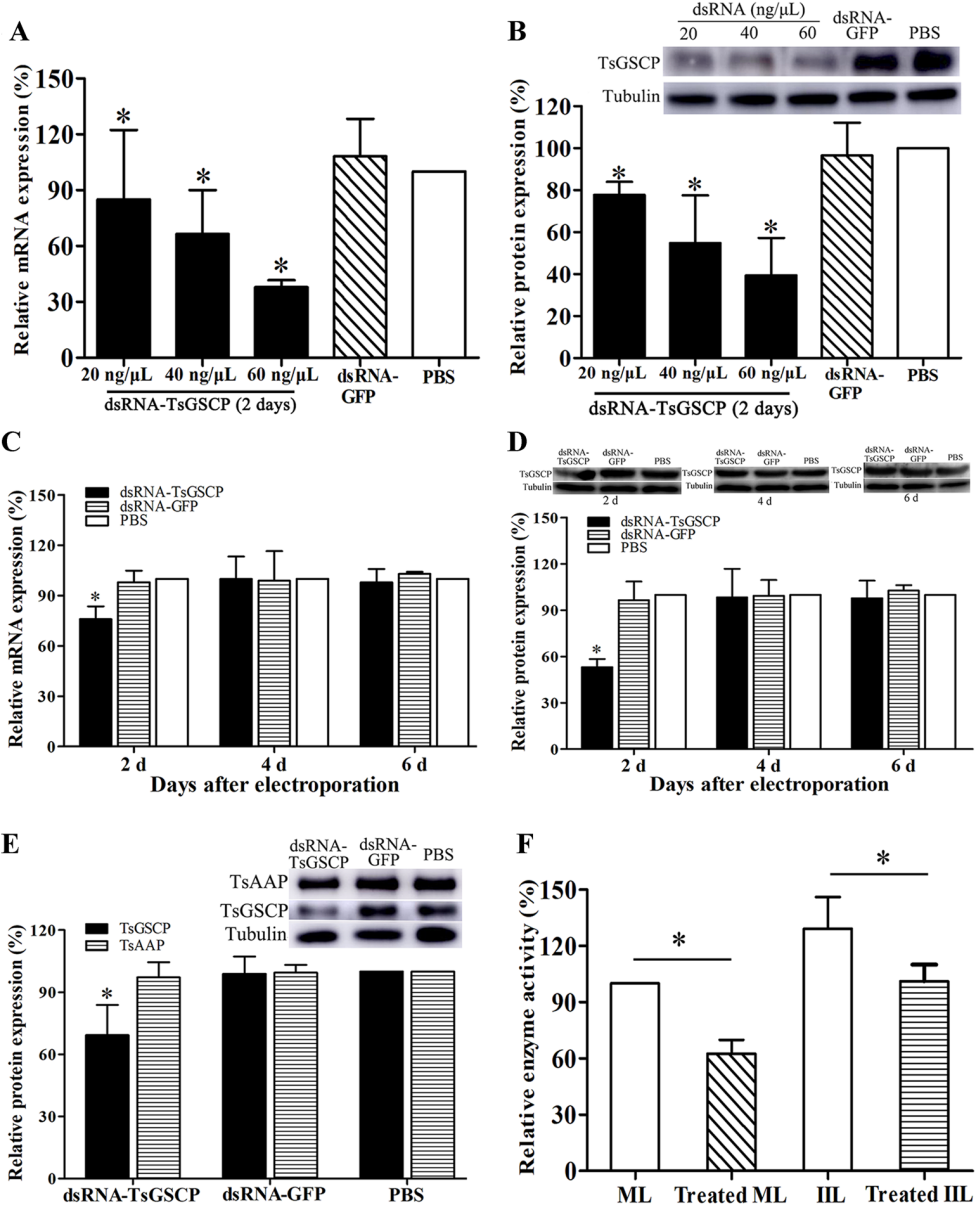
**Suppression of TsGSCP expression and enzymatic activity after dsRNA interference. A** Various doses of dsRNA-TsGSCP inhibited TsGSCP transcription. **B** Various doses of dsRNA-TsGSCP inhibited TsGSCP expression. **C** TsGSCP transcription level on different days after dsRNA transfection. **D** TsGSCP expression level as measured on different days after dsRNA transfection. **E** Expression levels of TsGSCP and *T. spiralis* aspartyl aminopeptidase (TsAAP) in ML treated using TsGSCP-specific dsRNA. **F** The ability of TsGSCP to enzymatically cleave substrate Z-FR-AMC was significantly reduced in dsRNA-TsGSCP-treated ML and IIL. *P < 0.05 relative to the PBS group.

After transfection with 40 ng/μL dsRNA and cultured for 2 days, the survival of the dsRNA-TsGSCP, dsRNA-GFP and PBS larva groups was 99.60, 99.44 and 99.63%, respectively (χ^2^ = 2.007, *P* > 0.05), indicating that electroporation has no evident effect on worm survival. When the ML were treated with 20, 40 and 60 ng/μL dsRNA and cultivated for 2 days, TsGSCP transcription levels were decreased by 14.82, 33.30 and 62.13%, respectively, compared to those in the PBS group (*P* < 0.05), and TsGSCP expression was reduced by 22.24, 45.28 and 60.51% (*P* < 0.05) (Figures 11A and B). Two, four and 6 days after treatment with 40 ng/μL dsRNA-TsGSCP, TsGSCP transcription levels were reduced by 24.25, 2.21 and 1.89%, respectively (*P* < 0.05), and TsGSCP expression was suppressed by 46.82, 1.73 and 2.26%, respectively (*P* < 0.05) (Figures 11C and D). However, TsGSCP expression levels were not suppressed when the worms were transfected with control dsRNA-GFP. Moreover, no obvious change in TsAAP expression was observed in ML treated with dsRNA-TsGSCP (Figure 11E), demonstrating that the dsRNA is TsGSCP-specific.

The results of the enzymatic activity assay showed that the natural TsGSCP activity among the crude proteins of dsRNA-treated ML and IIL was reduced by 37.39 and 28.00%, respectively, compared with untreated ML or IIL (χ^2^_ML_ = 45.399 and χ^2^_IIL_ = 23.464, *P* < 0.05) (Figure 11F).

### dsRNA-TsGSCP impaired larval infectivity, growth and fecundity

**Figure 12:**
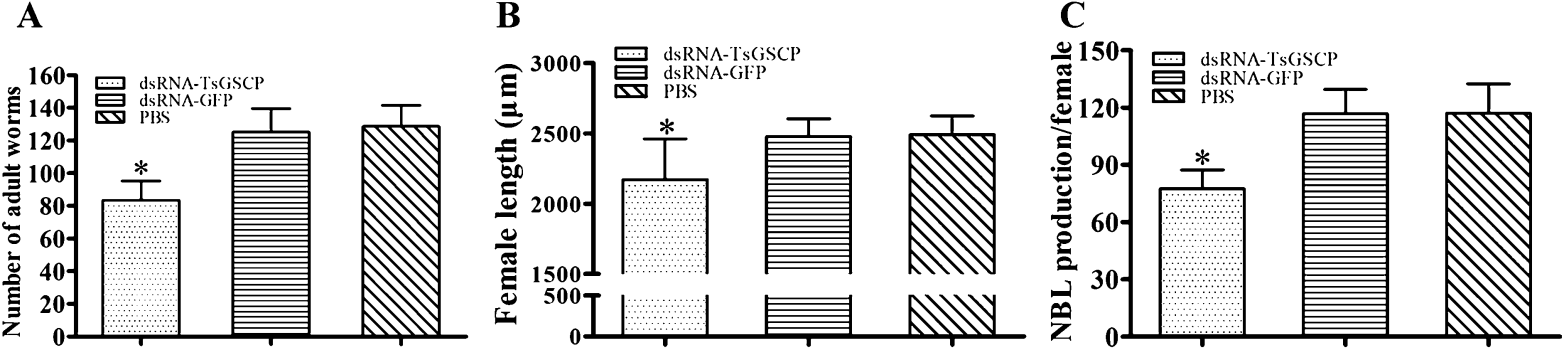
**Intestinal adult worm burden and female length and fecundity in mice infected with larvae treated with TsGSCP dsRNA. A** Intestinal adult worm burden (*n* = 10). **B** Adult female length (*n* = 25). **C** Female fecundity (*n* = 30). The data are shown as the means ± SD of the dsRNA-TsGSCP, control dsRNA-GFP and PBS groups. *P < 0.001 relative to the dsRNA-GFP control and PBS groups.

The number of 5 dpi adult worms in mice challenged with dsRNA-TsGSCP-transfected ML was 35.15% less than that in the PBS group (*F* = 37.374 *P* < 0.001), and the control dsRNA-GFP group did not show any decrease in adult worm burden (*P* > 0.05) (Figure 12A). The adult female length in the dsRNA-TsGSCP group was significantly shorter than that in the control dsRNA and PBS groups (*F* = 21.082, *P* ˂ 0.001) (Figure 12B), but male length showed no apparent change. Female fecundity in the dsRNA-TsGSCP transfection group worms was also evidently lower than that of the PBS group worms (*F* = 18.728, *P* < 0.001), but there were no significant differences in female fecundity between the control dsRNA-GFP and PBS groups (*P* > 0.05) (Figure 12C), demonstrating that silencing of the TsGSCP gene significantly impaired larval infectivity, development and survival and profoundly reduced female fertility.

## Discussion

Cysteine proteases usually consist of the enzyme precursor sequence and mature enzyme domain. They are usually nonenzymatic in the form of proenzymes but can self-hydrolyse as precursor peptides under acidic conditions and become catalytic enzymes [63]. Cysteine proteases have been extensively studied, and cathepsin B has been found to function either as an endopeptidase cleaving internal peptide bonds or as an exopeptidase (with carboxydipeptidase activity) acting on the end of the peptide chain [64]. Many studies on parasites have shown that cysteine protease may be the key to immune escape, encystation/excystation, moulting, and tissue cell invasion of parasites [31]. The mRNA encoding cathepsin B-like cysteine proteases (CATBs) is highly expressed in the genome of haemophagous nematodes, and the CATBs of hookworm (*Necator americanus*) are mainly localized in intestinal tissue [65]. Previous studies have shown that cathepsin B- and L-like proteases facilitate helminth larva invasion into host tissues [29]. A cathepsin B-like protease (Ac-cathB-1) is involved in host enteral epithelium invasion by *Angiostrongylus cantonensis*; incubation of rat intestine with Ac-cathB-2 resulted in the detachment of epithelial cells, and antiserum reduced L3 larva penetration [66]. Our previous study indicated that recombinant *T. spiralis* cathepsin B (rTsCB) facilitated larva penetration into IECs, whereas anti-rTsCB antibodies and RNAi suppressed larva penetration into enterocytes, suggesting that TsCB participated in larva invasion into the gut epithelium and might be a molecular target against *T. spiralis* intrusion [24].

In the present study, bioinformatics analyses showed that TsGSCP has a functional cathepsin B domain; cathepsin B belongs to the C1A peptidase family. TsGSCP was cloned into the pMAL-c2x expression vector and expressed in an *E. coli* expression system. Following purification, rTsGSCP was used to immunize mice and produce anti-rTsGSCP antibodies. The anti-rTsGSCP IgG titre was 10^5^ two weeks after the final immunization, which indicated that rTsGSCP has strong immunogenicity. In the Western blotting analysis, rTsGSCP was recognized by anti-rTsGSCP serum and infection serum. As shown in Figure 3C, several native TsGSCPs of 35.2–61.6 kDa among IIL soluble proteins were identified with anti-rTsGSCP serum but was not found among IIL ES proteins, suggesting that TsGSCP is a somatic worm protein, not secretory protein. The presence of several native TsGSCPs among IIL somatic proteins might be a result of different TsGSCP isoforms or post-translational modification and processing. It is likely that, as a member of the *T. spiralis* cathepsin B family, TsGSCP has the same antigenic epitopes as cathepsin B [26, 42, 49, 67].

The qPCR and Western blot analysis results showed that TsGSCP mRNA and protein expression at the IIL stage was higher than that at other developmental stages in the *T. spiralis* lifecycle. The results of the IIF analysis with intact worms showed native TsGSCP on the outer cuticle of intestinal IIL, AW and NBL but not on that of ML. As identified by immunohistological staining, native TsGSCP was principally located at the cuticle and intrauterine embryos of the nematode. The expression of TsGSCP in all lifecycles of the nematode suggested that this protease participates in *T. spiralis* growth, development and survival in the host [16, 41]. Moreover, native TsGSCP on the outer cuticle of intestinal IIL the first exposure of the parasite to the enteral milieu of the host, where it is contacted and recognized by IECs; hence, TsGSCP might be a key intrusion-related protease in the IIL stage [10, 26, 36]. In this work, the enzymatic activity of rTsGSCP, which hydrolysed the cathepsin substrate, was not observed (data not shown). In the present study, the enzymatic activity of rTsGSCP was assessed by using gelatine zymography and cysteine protease-specific substrate (Z-FR-AMC). Unfortunately, rTsGSCP did not degrade gelatine or the substrate (data not shown), indicating that rTsGSCP does not have the enzymatic activity of native cysteine protease. The absence of native cysteine protease activity by rTsGSCP is likely a result of improper folding form of rTsGSCP in *E. coli* [22]. Therefore, to obtain rTsGSCP with enzymatic activity, a eukaryotic expression system needs to be used or native TsGSCP needs to be isolated from *T. spiralis* larvae.

Protein–protein interactions between rTsGSCP and IECs were also investigated in this study. The far-Western blot and ELISA results showed that rTsGSCP specifically bound to IEC proteins in a dose-dependent manner. The IIF analysis results indicated that the binding sites of rTsGSCP and IECs were located in the cytoplasm. Previous studies have shown that when IIL were incubated with IECs, some proteases produced by IIL entered the IECs [18, 47]. The in vitro larval intrusion test showed that rTsGSCP markedly accelerated the worm intrusion into IECs, whereas anti-rTsGSCP antibodies profoundly impeded intrusion, and this acceleration and prevention was dependent on the dose of rTsGSCP and anti-rTsGSCP antibodies, respectively. The accelerated intrusion might be due to the specific binding of TsGSCP with IECs [22, 24]. The suppressive function of anti-rTsGSCP antibodies on larval intrusion into IECs might be a result of the cap-like antigen–antibody complex formed by TsGSCP and anti-TsGSCP antibodies at the larval anterior, which physically encloses the larval sensory receptors and interdicts the direct contact between IIL and the gut epithelium, impeding larval intrusion and penetration [16, 68]. However, it is necessary to characterize the IEC proteins that bind with TsGSCP using coimmunoprecipitation/mass spectrometry in further investigations.

In the parasitic nematode *T. spiralis*, RNAi is the primary technique used to investigate gene function [59]. To verify the biological roles of TsGSCP in the intrusion in this study, growth and survival of this nematode, TsGSCP-specific dsRNA was transfected into ML via electroporation. The results showed that after the ML were treated with 40 ng/μL dsRNA and cultured for 2 days, TsGSCP mRNA and protein expression was reduced by 24.25 and 46.82%, respectively. Natural TsGSCP activity in dsRNA-treated ML and IIL was inhibited by 37.39 and 28.00%, respectively. The number of dsRNA-treated larvae intruding into IECs in vitro was decreased by 25.02% compared to that of the control group. These results indicated that silencing of the TsGSCP gene by a specific dsRNA significantly decreased TsGSCP expression and enzymatic activity in larvae and dramatically suppressed the ability of larvae to intrude into IECs. The results further verified that TsGSCP facilitates gut epithelium intrusion by *T. spiralis* [21]. Moreover, an animal challenge experiment revealed a 35.15% reduction in enteral adult worm burden in mice 5 dpi with dsRNA-transfected ML. Enteral worm growth and female fertility were also obviously impaired by TsGSCP-specific dsRNA, as demonstrated by shorter adults and lower female reproductive capacity. In a previous study, silencing cysteine protease genes (FhCatB1 and FhCatL1) in newly excysted juvenile *Fasciola hepatica* by RNAi reduced gut penetration [69]. In another study, *Schistosoma japonicum* cathepsin B2 promoted skin invasion, while anti-rsjCB2 IgG clearly inhibited cercariae invasion into the skin [70]. These findings indicated that TsGSCP exerts a principal role in gut mucosal invasion, larval growth and female fecundity during *T. spiralis* infection [22].

In conclusion, TsGSCP was highly expressed at gut invasive IIL stages of the *T. spiralis* lifecycle and primarily localized to the cuticle and intrauterine embryos of this parasite. The rTsGSCP showed good immunogenicity. rTsGSCP exhibited the ability to specifically bind with IECs, and the binding site is within the cytoplasm of the IECs. rTsGSCP accelerated larval intrusion into IECs, whereas anti-rTsGSCP antibodies impeded larval intrusion; the acceleration/inhibition effect was dose-dependent for rTsGSCP/anti-rTsGSCP antibodies. Silencing of the TsGSCP gene by specific dsRNA significantly decreased TsGSCP expression and TsGSCP ability to hydrolyse the cysteine protease substrate Z-FR-AMC; RNAi also impaired larval invasive ability, growth and fecundity. These findings demonstrated that TsGSCP plays a principal role in gut intrusion, worm development and fecundity during the *T. spiralis* lifecycle and might be a candidate target for vaccine development against *Trichinella* intrusion and infection.

## Abbreviations

AWs: adult worms
DAB: 3,3′-Diaminobenzidine tetrahydrochloride
DAPI: 4′,6-Diamidino-2-phenylindole
ES: excretory/secretory
HRP: horseradish peroxidase
IIF: indirect immunofluorescence
IIL: intestinal infective larvae
IPTG: Isopropyl β-d-1-thiogalactopyranoside
ML: muscle larvae
NBL: new-born larvae
NC: nitrocellulose
OD: optical density
PBS: phosphate-buffered saline
rTsGSCP: recombinant *T. spiralis* gut-specific cysteine protease
TBST: Tris-buffered saline containing Tween
